# Special Issue “G Protein-Coupled Receptors: Molecular Mechanisms Involved in Receptor Activation and Selectivity”

**DOI:** 10.3390/life13010166

**Published:** 2023-01-06

**Authors:** Rossella Miele, Roberta Lattanzi

**Affiliations:** 1 Department of Biochemical Sciences “A. Rossi Fanelli”, CNR-Institute of Molecular Biology and Pathology, Sapienza University of Rome, Piazzale Aldo Moro 5, I-00185 Rome, Italy; 2 Department of Physiology and Pharmacology “Vittorio Erspamer”, Sapienza University of Rome, Piazzale Aldo Moro 5, I-00185 Rome, Italy

Welcome to the Special Issue of Life entitled “G Protein-Coupled Receptors: Molecular Mechanisms in Receptor Activation and Selectivity”. G protein-coupled receptors (GPCRs) are seven-transmembrane receptors that, when activated, transmit their signals predominantly through the alpha subunits of heterotrimeric G proteins. GPCRs are present in all cell types and regulate a variety of physiological functions, alterations of which can lead to pathogenic effects.

The purpose of this collection is to focus on biochemical, pharmacological, and structural evidence for the molecular mechanisms of GPCR activation, understanding of which is critical for the development of new highly specific drugs with fewer side effects.

The review presented by the editors in this issue summarizes the key cellular and biochemical mechanisms that regulate the prokineticin pathway. These include, as with other chemokines, genetic polymorphisms, modulation of mRNA splicing, regulation of expression at the transcriptional and posttranscriptional levels of prokineticins, and prokineticin receptors [1]. Prokineticin 1 (PK1) and prokineticin 2 (PK2), a new class of chemokine-like peptides, bind to the G protein-coupled receptors PKR1 and PKR2. The prokineticin signaling pathway promotes chemotaxis and the production of pro-inflammatory cytokines, the dysregulation of which leads to many pathological conditions such as cancer, pain, neuroinflammation, and neurodegenerative diseases such as Alzheimer’s and Parkinson’s disease [2,3].

The editors also describe the identification of a new alternatively spliced product of the pk2 gene encoding PK2, designated PK2C, that can bind and activate both prokineticin receptors. This new peptide was characterized in vitro by GST pull-down experiments and by photoactivatable cross-linking, and in vivo by nociceptive experiments, which showed that PK2C elicited strong sensitization of peripheral nociceptors to painful stimuli [4].

Because melanocortin-4 receptor mutations predominantly cause monogenic obesity, in the article by Hoepfner et al., the authors characterized five melanocortin-4 receptor (MC4R) nonsense mutants (W16X, Y35X_D37V, E61X, W258X, Q307X) by using aminoglycoside-mediated translational bridging to overcome stop mutations. In transfected HEK-293 cells, they tested whether translational bridging by the aminoglycoside geneticin in combination with the high-affinity ligand setmelanotide, which is effective in patients with proopiomelanocortin or leptin receptor deficiency, is a treatment option for affected patients. The authors concluded that N-terminal mutants were only slightly expressed regardless of treatment with geneticin, whereas mutants with nonsense mutations in transmembrane helix 6 or helix 8 showed wild-type-like expression [5].

In the article by Colucci et al., the authors examine the possible role of FPR2/ALX in nociception in mice. Formyl peptide receptor type 2 (FPR2/ALX) is a member of the formyl peptide receptor (FPR) family, a member of class A G protein-coupled receptors (GPCRs). The authors demonstrated a possible role for FPR2/ALX in pain control. Indeed, intrathecal administration of the formyl peptide receptor type 1 (FPR1) agonist fMLF and the FPR2/ALX agonist BML-111 reduced nociception, and these effects were reduced by concomitant administration of the FPR2/ALX antagonist WRW4. Furthermore, measurement of cytokines and brain-derived neurotrophic factor (BDNF) in the spinal cords of neuropathic mice suggested that the antinociceptive effects of BML-111 may depend on the reduction of cytokine release and BDNF in the spinal cord [6].

In the article by Teleuca et al., the authors used an in vivo method to assess polyphosphoinositide hydrolysis (PI) to investigate whether spatial learning and memory extinction cause changes in signaling of the metabotropic glutamate receptor mGlu5 in the hippocampus and prefrontal cortex. The results provide evidence for a role of mGlu5 receptors in the mechanisms underlying spatial learning and suggest that mGlu5 receptors are potential drug targets in disorders wherein cognitive functions are impaired or aversive memories are inappropriately retained [7].

Adipokinetic hormone (AKH) is one of the major metabolic neuropeptides in insects and acts similarly to glucagon in vertebrates. AKH exerts its function by binding to a rhodopsin-like G protein-coupled receptor located in the cell membrane of the fat body.

In the study by Jackson et al., an in silico screen was performed to identify compounds that can bind AKHR of *S. gregaria*. Among the seven docked compounds, compounds were selected whose binding energy was sufficient to compete with the endogenous ligand. One of the ligands, ZINC000257251537, was tested in a homospecific biological in vivo assay and showed significant antagonistic activity [8].

Schwann cells (SCs) express cholinergic receptors, suggesting a role for cholinergic signaling in controlling SC proliferation, differentiation, and/or myelination. In the study by Botticelli et al., the signal transduction pathways activated by the orthosteric M2 agonist arecaidine propargylester (APE) in SCs were investigated. The data obtained showed that activation of the M2 receptor, in addition to the canonical Gi protein-coupled signaling pathway, modulates non-canonical signaling pathways involving the mTORC1 complex and other kinases, whose activation may contribute to the inhibition of SC proliferation and migration and address SC differentiation [9].

In the article of Dasgupta et al., the pharmacological activity of veldoreotide, a somatostatin analogue, on somatostatin receptors 2,4,5 (SSTR 2,4,5) was investigated. The results showed that veldoreotide inhibited cell proliferation in BON-1 cells expressing SSTR4 to a greater extent than somatostatin SS-14 and to a similar extent as the SSTR4 agonist J-2156 in the presence of SSTR2 and SSTR5 antagonists. Veldoreotide is a complete agonist of SSTR2, SSTR4, and SSTR5 [10].

Trait anxiety is a susceptible personality factor for anxiety and depression. There is evidence that 5-hydroxytryptamine receptor 1B (5-HT1B) gene polymorphisms play an important role in emotional disorders. Ruan et al. performed genotyping for four single nucleotide polymorphisms (SNP) in 388 high-anxiety (HTA) and 463 low-anxiety (LTA) individuals in Chinese Han high schools. The results suggest that 5-HT1B rs13212014 may play a role in trait anxiety and reduce the risk of trait anxiety. These results also provide a new insight into the molecular mechanism underlying trait anxiety [11].

Neuropathic pain (NP) is a type of chronic pain for which there has been no satisfactory treatment. Even strong opioids show poor efficacy and the paradoxical ability to increase pain sensitivity in NP patients. As a growing body of evidence suggests that chemokines are upregulated according to NP pathophysiology, this review by Vincenzi et al. examined the role of chemokines and chemokine receptors in the development and maintenance of NP. The authors concluded that potent opioids together with drugs blocking the specific chemokine–chemokine receptor axis might be the right compromise for a favorable risk–benefit ratio in the treatment of NP [12].
